# Catch Them Young! Changing Attitudes and Perspectives Towards Psychiatry by Using Patients as Educators Early in Medical Training

**DOI:** 10.1192/bjo.2022.114

**Published:** 2022-06-20

**Authors:** S Caroline Buck, Nicola Combs, Amina Rashid, Victoria Ozidu, Adeola Akinola

**Affiliations:** 1 Pennine Care NHS Foundation Trust, Greater Manchester, United Kingdom.; 2 University of Manchester, Manchester, United Kingdom; 3 Greater Manchester Mental Health NHS Foundation Trust, Greater Manchester, United Kingdom

## Abstract

**Aims:**

This teaching project aims to improve attitudes and perspectives towards psychiatry by using Patient as Educators (PaE) in a psychiatry teaching program early in medical training.

**Methods:**

Following the success of a small pilot study in 2020, the project was rolled out to the entire second year medical student body in 2021. Two-hour interactive sessions were delivered online to groups of approximately twelve students. Each session was introduced by a psychiatrist, followed by PaE discussion with questions and answers. The students completed a bespoke online survey at the beginning and the end of the session, looking at attitudes towards psychiatry. Comparative analysis of attitudes pre- and post-intervention was then undertaken. Qualitative data were examined through content analysis and quantitative methods were used to compare pre- and post- attitudes on the Likert scale.

**Results:**

The pre- and post-intervention questionnaires were completed by 373 and 305 students respectively. Both pre- and post-intervention attitudes were overwhelmingly positive. Post-intervention qualitative results demonstrate the session, especially the PaE, helped students to better understand the complexities of mental illness, the stigma faced and the potential efficacy of good treatment. There was a 25.7% increase in students’ perception of preparedness to see mentally unwell patients. The most significant findings were that the majority of students found having the PaE valuable in improving attitudes regarding the value of psychiatry (72.8% agreed/strongly agreed) and increasing interest in the speciality (84% agreed/strongly agreed).

**Conclusion:**

Early experience to clinical placements is an essential component in medical education. In Psychiatry, apart from gentle introduction into the specialty, it is essential that students are orientated into the world of mental health and its various challenges. This project has clearly demonstrated the effectiveness of early exposure of medical students to psychiatry as a specialty. It also demonstrates the effectiveness of using PaE in medical education. Further research would aim to examine whether effect on attitudes persist and correlate the effect on early exposure on recruitment to the speciality.

